# Temporal trends of suicidality among adolescents in the last decade in Piedmont, Northwestern Italy: Michela Bersia

**DOI:** 10.1093/eurpub/ckac131.219

**Published:** 2022-10-25

**Authors:** P Berchialla, M Bersia, E Koumantakis, E Ferracin, RI Comoretto, L Charrier, P Dalmasso

**Affiliations:** 1 Clinical and Biological Science, University of Torino, Orbassano, Turin, Italy; 2 Sciences of Public Health and Pediatrics, University of Torino, Turin, Italy; 3 Epidemiology Service, ASL TO3 Piedmont Region, Grugliasco, Turin, Italy; 4 Post Graduate School of Medical Statistics, University of Torino, Turin, Italy

## Abstract

**Background:**

Over the last decade, trends of suicidality among adolescents remain unclear. We conducted a cross-sectional study using surveillance data collected over the past 10 years in the Piedmont region, Italy, to explore temporal trends in suicidality among hospitalized adolescents.

**Methods:**

This was a retrospective study over an 11-year period of time. The target cohort was adolescents aged 13-19 discharged from inpatient/day-hospital care with at least one suicidality-related ICD9-CM code (i.e., suicidal ideation-SI, suicidal risk-SR and suspected suicide-SS) between 2011 and 2021 in Piedmont (Northwestern Italy). Social-economic related data is available from Census 2011. Yearly incidence rates (IR) were calculated based on the overall hospitals’ catchment population and by sex. Poisson regression model was estimated to evaluate the trend over time and the association with sex, and a potential effect of the COVID-19 pandemic. A non-linear trend was allowed by modelling natural splines.

**Results:**

We included 490 adolescents (median age: 15 years, IQR: 13-16), 380 girls and 110 boys, with ICD9-CM codes for SI (264; 53.9%), SR (142; 29%), SS (90; 18.4%) at first discharge. Girls showed a higher risk of repeated inpatient care than boys (19.2% vs 7.3%, p < 0.01). Since 2013, yearly suicidality IRs started increasing linearly in boys (+1.7/100,000 per year, 95%CI: 0.7-2.8). Apparently, suicidality IRs increasing in girls were observed since 2011 (+5.8/100,000 per year, 95%CI 2.8-8.9) and were significantly higher than in boys (p < 0.001).

**Conclusions:**

Suicidality among young inpatients increased in Piedmont during the last decade. Females seemed to be more affected than males. Further research is needed to better understand gender-related risk factors for suicidality.

**Key messages:**

• There has been an increase in the number of adolescents reporting suicidal ideation in the last decade, especially in girls.

• Intervention strategies are urgently needed to reverse a potentially alarming trend in suicidality among young people.

